# Biliary Reconstruction in Liver Transplantation for Primary Sclerosing Cholangitis: Outcomes of an Anatomy-Driven Shift Towards Duct-to-Duct Anastomosis

**DOI:** 10.3390/jcm15155871

**Published:** 2026-07-27

**Authors:** Felix Becker, Felicia Kneifel, Janna-Maria Keßling, Shadi Katou, Haluk Morgül, Andreas Pascher, Philipp Houben

**Affiliations:** Department of General, Visceral and Transplant Surgery, University Hospital Muenster, 48149 Muenster, Germany

**Keywords:** PSC, liver transplantation, biliary reconstruction

## Abstract

**Background/Objectives**: Selecting the optimal biliary reconstruction technique for liver transplantation (LT) in primary sclerosing cholangitis (PSC) remains challenging. Many centers have historically favored Roux-en-Y hepaticojejunostomy (RYHJ) to bypass potentially diseased recipient ducts, whereas duct-to-duct (D-D) anastomosis preserves endoscopic access and decreases cholangitis rates. We evaluated outcomes during an institutional, anatomy-driven transition toward D-D reconstruction. **Methods**: We retrospectively analyzed primary LTs for PSC performed at Münster University Hospital between January 2008 and February 2021. Patients were stratified by biliary reconstruction technique into a RYHJ and D-D group. The reconstruction strategy was based on the recipient’s bile duct anatomy (D-D when distal extrahepatic duct pathology was absent and duct quality was deemed suitable; RYHJ otherwise). Postoperative complications within 12 months, biliary complications, and long-term patient and graft survival were assessed. **Results**: Forty-six patients were included (RYHJ n = 24; D-D n = 22). Overall biliary complication rates and patient and graft survival were comparable between the groups. Anastomotic strictures occurred more frequently after D-D reconstruction, whereas the rates of bile leakage, cholangitis, and revision surgery were similar between the two groups. In exploratory multivariable analyses, reconstruction type was not associated with mortality or overall biliary complications, whereas recipient age and male donor sex were associated with biliary complications. **Conclusions**: In selected patients with PSC undergoing LT with an anatomy-driven reconstruction strategy, D-D anastomosis achieved outcomes comparable to those of RYHJ, with a higher rate of anastomotic strictures but preserved potential for endoscopic management.

## 1. Introduction

Primary sclerosing cholangitis (PSC) is an immune-mediated, chronic inflammatory, and cholestatic disease that affects the intrahepatic and extrahepatic bile ducts. The phenotypic hallmark of PSC is the multifocal formation of fibrotic strictures in the biliary tree. PSC typically progresses slowly from asymptomatic cholestasis to recurrent cholangitis, secondary biliary cirrhosis, and ultimately end-stage liver disease [1,2]. Although considered rare, with an annual pooled global incidence rate of 0.87 per 100,000 person-years [3], PSC is a leading indication for liver transplantation (LT), accounting for approximately 5–15% of all LTs in Europe and North America [1,4]. Approximately 40% of patients with PSC require LT in their lifetime [4], with a median transplant-free survival of 14.5 years [5].

Patients undergoing LT for PSC are at a significantly higher risk of post-transplant biliary complications, including anastomotic and non-anastomotic strictures, bile leakage, and recurrent PSC (rPSC), than recipients undergoing transplantation for other etiologies. This increased susceptibility is likely to be multifactorial and involves pre-existing biliary tract inflammation, altered ductal anatomy, and immunological mechanisms [6,7,8].

Consequently, biliary reconstruction is a central technical determinant of early morbidity and long-term graft function in this population. Two main techniques are commonly used: Roux-en-Y hepaticojejunostomy (RYHJ), a biliodigestive anastomosis (BDA), and duct-to-duct anastomosis (D-D). Traditionally, RYHJ has been the standard approach because it bypasses the recipient distal bile duct and may be favored when the distal duct quality is poor. However, it eliminates the regulatory function of the major duodenal papilla, which normally prevents retrograde contamination of the bile ducts. Therefore, RYHJ is associated with an increased risk of recurrent cholangitis. Moreover, endoscopic access to the biliary system is limited because of altered intestinal anatomy, which complicates postoperative diagnostic and therapeutic interventions [9]. In contrast, D-D anastomosis preserves the natural anatomy and function of the sphincter of Oddi, offering better endoscopic accessibility in the event of postoperative complications and a potentially lower risk of ascending cholangitis [10]. However, concerns persist regarding the risk of biliary strictures, predilection for recurrence at the anastomosis site, and risk of cholangiocarcinoma in the remaining recipient bile duct when performing D-D anastomoses in patients with PSC [2,11,12].

Contemporary evidence suggests that reconstruction should be individualized, and current guidelines support D-D reconstruction when the recipient’s distal bile duct is free of relevant PSC involvement or dysplasia [9,10,11]. These recommendations are based on preoperative biliary imaging using magnetic resonance cholangiopancreatography (MRCP) or endoscopic retrograde cholangiopancreatography (ERCP), ideally with brush cytology, as well as intraoperative inspection, palpation, probing, and, in selected cases, frozen section evaluation of the recipient bile duct [12].

In recent years, our center has adopted a structured, anatomy-driven approach with an increasing use of D-D reconstruction when distal duct pathology was absent, and duct quality is suitable. Although the feasibility of D-to-D reconstruction in selected PSC recipients has been supported by previous studies and meta-analyses, less is known about the real-world implementation and clinical consequences of an anatomy-driven transition from routine RYHJ toward selective D-D reconstruction within a single institutional practice [10,13,14]. The present study therefore does not aim to redefine the general standard of biliary reconstruction in PSC, but rather to evaluate the clinical consequences of this institutional selection strategy over time.

We aimed to describe the pattern of biliary complications under an anatomy-based selection strategy and assess whether clinically relevant differences in outcomes were observed between reconstruction approaches. The primary endpoint was the incidence of biliary complications within 12 months after LT, with secondary endpoints including overall postoperative morbidity, cholangitis, stricture phenotypes, biliary interventions, and long-term patient and graft survival.

## 2. Materials and Methods

### 2.1. Study Design and Study Population

This retrospective, single-center cohort study included patients with PSC who underwent their first LT at the Department of General, Visceral, and Transplant Surgery, University Hospital Münster. All patients with confirmed PSC who underwent LT between January 2008, and February 2021 were screened for inclusion. Patients aged <18 years, those undergoing combined or multivisceral transplantation, and those who underwent re-transplantation were excluded, except when re-transplantation occurred within the first year post-LT and was considered a complication. All patients had a minimum follow-up period of 12 months. This study was approved by the local Ethics Committee (Approval No. 2022-335-f-S).

### 2.2. Biliary Reconstruction Strategy

**Figure 1:**
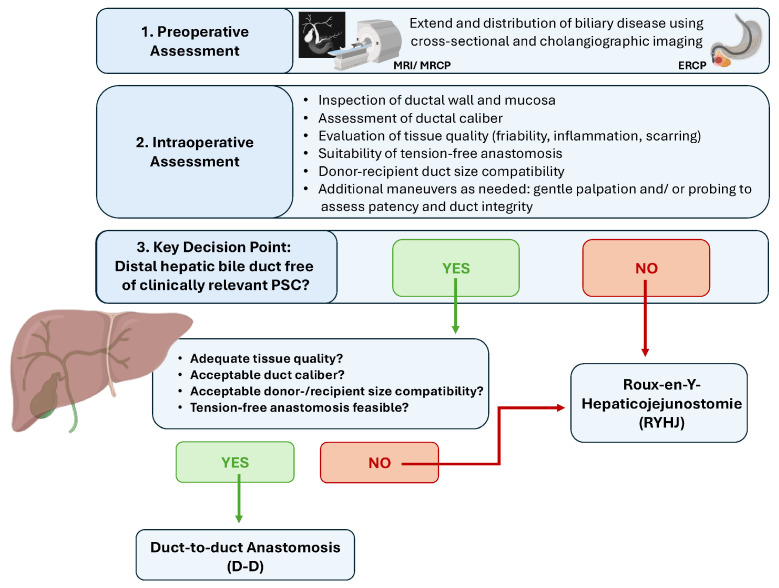
**Institutional decision-making algorithm for biliary reconstruction in liver transplantation for primary sclerosing cholangitis**.

Patients were stratified into two groups based on the type of biliary reconstruction: (1) RYHJ and (2) D-D anastomosis. The biliary reconstruction technique was selected according to an institutional anatomy-driven approach based on the preoperative evaluation of recipient bile duct involvement and intraoperative assessment of duct quality. Preoperatively, the extent and distribution of biliary diseases were evaluated using cross-sectional and cholangiographic imaging, primarily MRCP and ERCP. Intraoperatively, the recipient’s extrahepatic bile duct was assessed by inspection of the ductal wall and mucosa, evaluation of tissue quality (e.g., friability, inflammation, scarring), donor–recipient duct size compatibility, and the feasibility of a tension-free anastomosis. Additional maneuvers, such as gentle palpation and/or probing, were performed as needed to assess duct patency and integrity. D-D reconstruction was performed when the distal extrahepatic bile duct was free of clinically relevant PSC involvement and intraoperative assessment confirmed favorable anatomy. No predefined bile duct diameter cutoff was applied. Instead, the choice of reconstruction was based on the overall anatomical and technical feasibility. Figure 1 illustrates the institutional decision-making algorithm for biliary reconstruction.

### 2.3. Data Collection

Data were extracted from electronic health records, the Orbis internal hospital system, and the Eurotransplant Network Information System (ENIS) and were anonymized prior to analysis. Recipient variables included age, sex, body mass index (BMI), Model for End-Stage Liver Disease (MELD) score at listing and transplantation, waiting time, number and timing of pre-transplant endoscopic procedures (ERCPs), peak bilirubin values (historical and perioperative), and imaging-based classification of bile duct involvement. Donor variables included age, sex, and BMI. The preoperative and intraoperative data included cold ischemia time (CIT), warm ischemia time (WIT), duration of surgery, and presence of a biliary stent at the time of LT. Comorbidities and relevant clinical history (inflammatory bowel disease (IBD), autoimmune hepatitis (AIH) overlap, prior colectomy, and history of cholangiocarcinoma) were recorded.

### 2.4. Outcome Measures

The primary endpoint was the incidence of biliary complications within 12 months of LT. Biliary complications included cholangitis, anastomotic stricture, and bile leakage. Cholangitis was defined as fever or clinical suspicion of infection in combination with cholestatic laboratory abnormalities or supportive imaging findings that required antimicrobial therapy. Anastomotic stricture was defined as anastomotic narrowing demonstrated by imaging and/or ERCP, with associated cholestasis and/or the need for endoscopic, percutaneous, or surgical intervention. Bile leak was defined as clinically relevant biliary leakage documented by drain characteristics/biochemical confirmation, imaging, endoscopic findings or reoperation.

Secondary outcomes included overall postoperative morbidity within 12 months, the Comprehensive Complication Index (CCI) during index hospitalization, intensive care unit (ICU) and total hospital length of stay, readmissions within 12 months, need for biliary interventions (ERCP, stent placement, percutaneous drainage, or surgical revision), retransplantation, rPSC, and patient and graft survival.

### 2.5. Statistical Analysis

Continuous variables are reported as mean and standard deviation (SD) or median with interquartile range (IQR), as appropriate. Categorical variables are presented as counts and percentages. Group comparisons were performed using Student’s *t*-test for approximately normally distributed variables and the Mann–Whitney U test otherwise. Categorical variables were compared using Fisher’s exact test. Associations with biliary complications were explored using logistic regression analysis. Given the sample size, multivariable models were limited to a small number of clinically selected covariates and are presented as exploratory. Time-to-event outcomes (patient survival, graft survival, and cholangitis-free survival) were analyzed using Kaplan–Meier methods and compared using log-rank testing. A two-sided *p*-value < 0.05 was considered statistically significant. Analyses were performed using IBM SPSS Statistics (version 29) and GraphPad Prism (version 9.3.1).

## 3. Results

### 3.1. Study Population Characteristics

**Figure 2:**
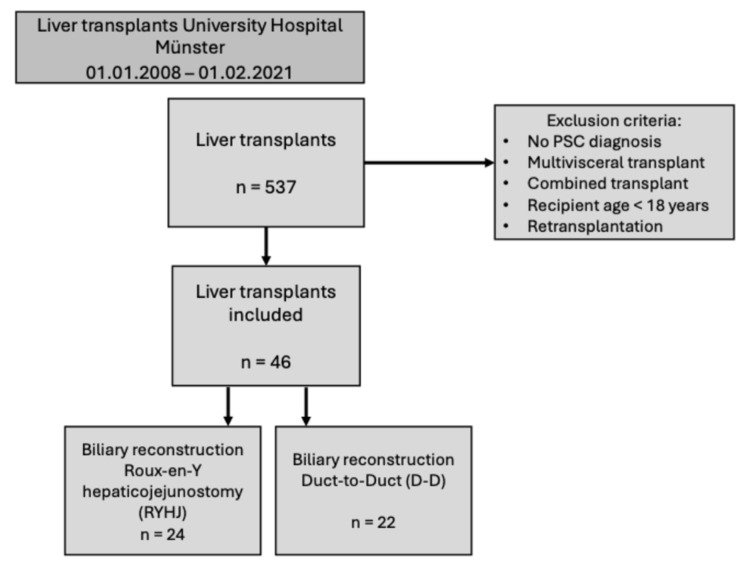
**Study flow diagram.** Flowchart of patient selection for this retrospective cohort study. Between January 2008 and February 2021, 537 liver transplantations (LTs) were screened at the University Hospital Münster. Of these recipients with primary sclerosing cholangitis (PSC) who underwent primary LT, 46 met the inclusion criteria and were analyzed. Patients were excluded for the absence of PSC, multivisceral or combined transplantation, recipient age < 18 years, or retransplantation as the index procedure. The included recipients were stratified by biliary reconstruction into Roux-en-Y hepaticojejunostomy (RYHJ; n = 24) and duct-to-duct (D-D) anastomosis (n = 22).

Between January 2008 and February 2021, 46 patients with PSC underwent primary LT at the University Hospital Münster. Biliary reconstruction was performed using RYHJ in 24 patients (52%) and D-D anastomosis in 22 patients (48%) (Figure 2).

**Figure 3:**
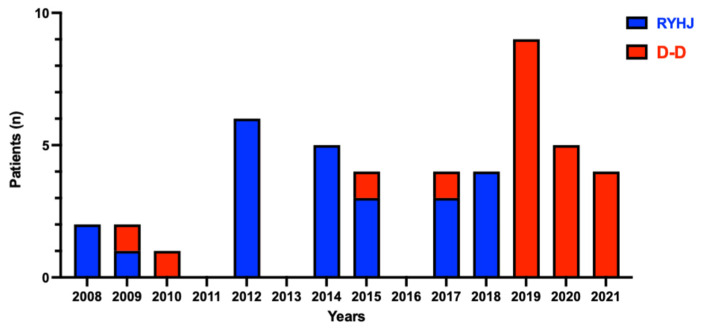
**Annual distribution of biliary reconstruction techniques.** Yearly distribution of biliary reconstruction techniques among patients with PSC undergoing primary LT between 2008 and 2021. Bars indicate the number of patients per year who underwent RYHJ (blue) or D-D reconstruction (red), illustrating the institutional transition toward an increased use of D-D reconstruction in later years. D-D, duct-to-duct; LT, liver transplantation; PSC, primary sclerosing cholangitis; RYHJ, Roux-en-Y hepaticojejunostomy.

RYHJ was the predominant technique in the earlier years of the study period, particularly before 2018. However, since 2019, a strategic shift in the surgical approach has occurred, with D-D anastomosis becoming the preferred reconstruction method at our tertiary referral center (Figure 3).

The donor and recipient baseline characteristics, including age, sex, and BMI, were comparable between the groups. There were no significant between-group differences in Eurotransplant Donor Risk Index (ET-DRI) (*p* = 0.561), MELD score (*p* = 0.941), or waiting time (*p* = 0.655). IBD was present in 70% of the recipients and did not differ significantly between the groups (RYHJ 79% vs. D-D 59%, *p* = 0.202). Pretransplant ERCP within one year was common (RYHJ 100% vs. D-D 86%, *p* = 0.101), and pretransplant biliary stent placement was more frequent in the RYHJ group (29% vs. 18%, *p* = 0.301). Endoscopic evaluation revealed a significantly higher rate of distal extrahepatic bile duct involvement in the RYHJ group than in the D-D group (46 vs. 14%; *p* = 0.026). Conversely, proximal hilar involvement was significantly more frequent in the D-D group (77% vs. 42%; *p* = 0.019). The intraoperative parameters, including CIT, WIT, and operative duration, were similar between the groups (Table 1).

### 3.2. Early Postoperative Course

The median ICU length of stay was four days in both groups (IQR RYHJ, 2–11 days vs. D-D, 2–5 days; *p* = 0.085), and the median hospital length of stay was comparable (31 vs. 29 days; *p* = 0.667). The CCI during the index hospitalization did not differ significantly (38 ± 29 vs. 43 ± 26; *p* = 0.545). The rates of reoperation (38% vs. 27%; *p* = 0.539), renal insufficiency (21% vs. 18%; *p* = 1.000), hemorrhage (33% vs. 27%; *p* = 0.484), and biopsy-proven acute rejection (25% vs. 41%; *p* = 0.348) were similar. Retransplantation within the first postoperative year occurred in two patients in each group (8% vs. 9%; *p* = 1.000) (Table 2).

### 3.3. Biliary Complications and Biliary Interventions

Cholangitis occurred in 12/24 (50%) RYHJ recipients and 9/22 (41%) D-D recipients within 12 months after LT (*p* = 0.568). Bile leakage occurred in 7/24 (29%) RYHJ recipients and 3/22 (14%) D-D recipients (*p* = 0.289). Anastomotic stricture/stenosis was observed only after D-D reconstruction (3/22; 14%), with no events in the RYHJ group (*p* = 0.101). Surgical revision of the biliary anastomosis was more frequent after RYHJ than after D-D reconstruction (29% vs. 14%; *p* = 0.289).

**Figure 4:**
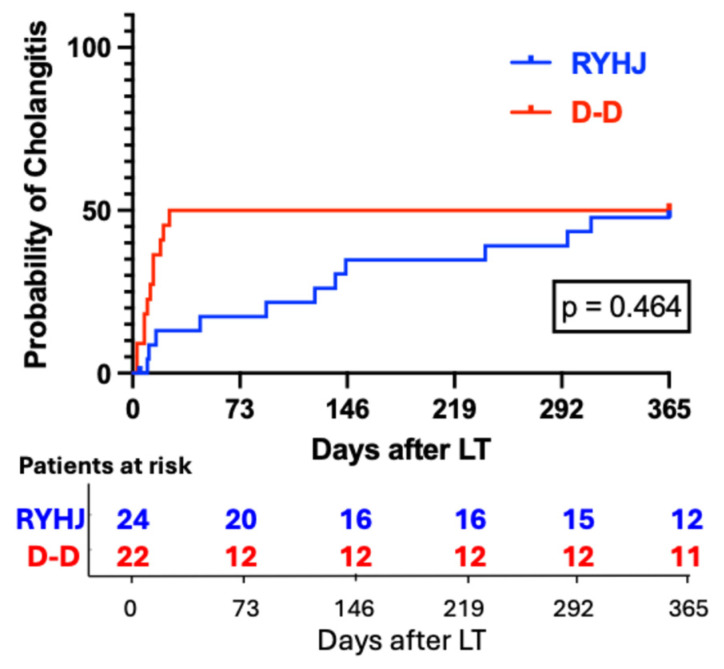
**Cholangitis-free survival and time to first cholangitis within 12 months after liver transplantation.** Kaplan–Meier analysis of time to first postoperative cholangitis within 365 days after LT, stratified by biliary reconstruction technique (RYHJ vs. D-D). Curves depict the cumulative probability of the first cholangitis episode during the follow-up; tick marks indicate censoring. The numbers at risk are shown below the *x*-axis. The groups were compared using the log-rank test. D-D, duct-to-duct; LT, liver transplantation; PSC, primary sclerosing cholangitis; RYHJ, Roux-en-Y hepaticojejunostomy.

The time to the first cholangitis episode did not differ significantly between the groups (log-rank *p* = 0.464); however, cholangitis tended to occur later in the postoperative course in the D-D group (Figure 4).

ERCP within the first postoperative year was more frequent after D-D reconstruction (RYHJ 29% vs. D-D 55%; *p* = 0.134). Biliary stent placement after LT occurred exclusively after D-D reconstruction (0% vs. 36%; *p* = 0.001), reflecting the greater feasibility of endoscopic access after D-D reconstruction. (Table 2).

One recipient in the RYHJ group was diagnosed with cholangiocarcinoma 3.5 years after transplantation, with metastatic disease at presentation, and died 8 months later. Recurrent PSC was documented in two patients (8%) in the RYHJ group and in no D-D recipients (*p* = 0.171) (Table 2).

### 3.4. Long-Term Graft and Patient Survival

**Figure 5:**
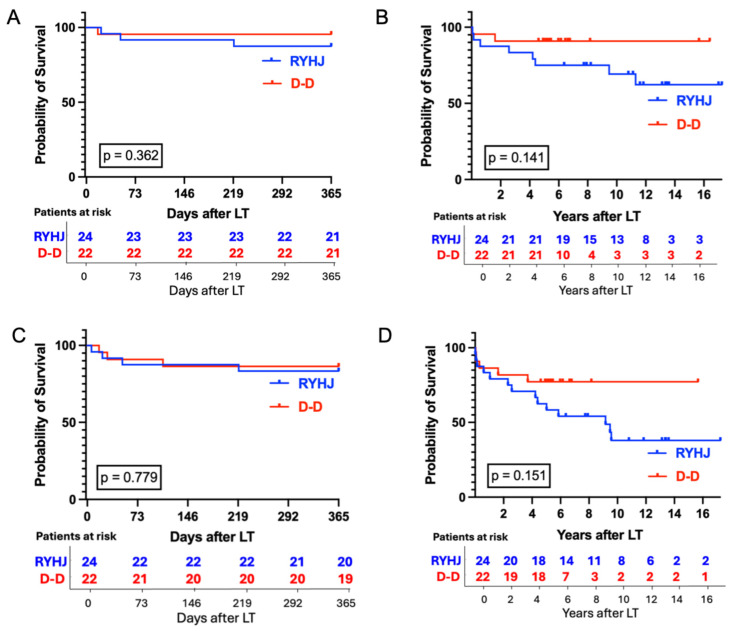

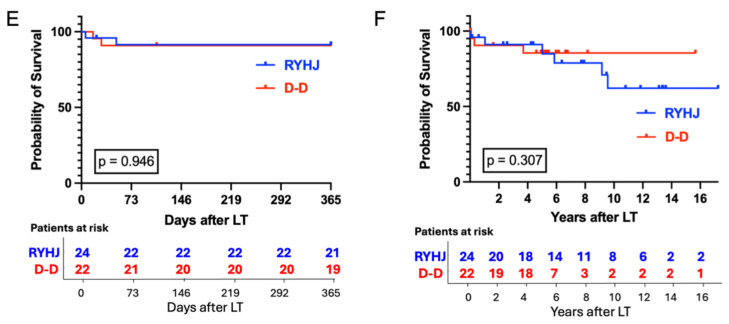
**Patient and graft survival after liver transplantation stratified by biliary reconstruction.** Kaplan–Meier survival analyses comparing outcomes after PSC liver transplantation between RYHJ and D-D reconstructions. Tick marks indicate censoring, and numbers at risk are shown below each plot. (**A**) 1-year patient survival, (**B**) overall patient survival, (**C**) 1-year graft survival, (**D**) overall graft survival, (**E**) 1-year death-censored graft survival, and (**F**) overall death-censored graft survival. D-D, duct-to-duct; LT, liver transplantation; PSC, primary sclerosing cholangitis; RYHJ, Roux-en-Y hepaticojejunostomy.

Patient survival at 90 days, 1 year, 3 years, and 5 years was 92%, 88%, 83%, and 75%, respectively, after RYHJ and 95%, 95%, 91%, and 91% after D-D reconstruction. The overall graft survival rates at the corresponding time points were 88%, 83%, 71%, and 63% after RYHJ and 91%, 86%, 82%, and 77% after D-D reconstruction. There were no statistically significant differences in patient survival or graft survival between the groups according to log-rank testing (Table 2; Figure 5).

### 3.5. Risk Factors for Biliary Complications

Exploratory logistic regression analysis was used to assess the impact of various parameters on biliary complications. The composite endpoints included cholangitis, anastomotic stricture, and/or anastomotic leakage. Older recipient age and male donor sex were associated with biliary complications (recipient age: OR 1.128, 95% CI 1.031–1.234, *p* = 0.008; donor sex: OR 11.118, 95% CI 1.516–81.538, *p* = 0.018). Reconstruction type was not associated with biliary complications (OR 1.015, 95% CI 0.318–3.244, *p* = 0.979) (Table 3).

## 4. Discussion

LT is the definitive treatment for advanced PSC; however, biliary complications and rPSC remain important determinants of morbidity and long-term graft function [1,4]. The optimal biliary reconstruction technique in this context remains controversial. Historically, RYHJ has been advocated as the standard reconstruction method for PSC to bypass potentially affected recipient ducts and mitigate concerns regarding persistent disease in the extrahepatic biliary tree. More recent data, including meta-analyses and contemporary single-center series, have challenged this dogma by showing no relevant differences in overall biliary stricture rates, graft survival, or rPSC between RYHJ and D-D reconstruction, provided that patients are appropriately selected [10,13,14]. In this retrospective PSC cohort study, an anatomy-driven transition toward D-D reconstruction was not associated with worse early postoperative morbidity or inferior long-term graft or patient survival. Importantly, the primary endpoint (biliary complications within 12 months) was comparable between the RYHJ and D-D reconstructions.

In addition, the 1-, 3-, and 5-year survival rates in this series fell within the range reported for PSC LT cohorts in contemporary reviews, underscoring that the institutional shift toward D-D did not compromise long-term outcomes [13,14,15,16]. These findings support the feasibility of a selective D-D approach in appropriately chosen PSC recipients. The absence of an independent effect of reconstruction type in the present study aligns with recent large-scale and meta-analytic data, which similarly failed to show a clear superiority of either D-D or RYHJ in terms of global biliary complication risk when accounting for confounders [10].

Nonetheless, the type and management of biliary complications differ between reconstruction strategies. Although our study was not powered to detect statistically significant differences because of the limited sample size, the distinct postoperative biliary complication profiles observed with the two reconstruction techniques are clinically relevant and may have important implications for surgical decision-making. RYHJ, while remaining the standard approach for complex biliary reconstruction, was associated with a higher incidence of bile leakage and a greater need for subsequent surgical revision of the biliary anastomosis in our cohort. This finding is consistent with reports suggesting that RYHJ anastomoses may carry a higher risk of leakage and ascending infection. In contrast, anastomotic strictures requiring endoscopic intervention were observed exclusively after D-D reconstruction. This was reflected by the higher frequency of postoperative ERCP and the exclusive use of biliary stents in this group, underscoring the clinical advantage of preserving endoscopic access for the minimally invasive diagnosis and management of biliary complications [10,14,17]. Given the chronic, relapsing nature of PSC and the potential for late biliary pathology, preservation of endoscopic access may be particularly relevant in younger recipients with a long post-transplant life expectancy [6,11,15,18].

rPSC is a key determinant of long-term outcomes after LT. In a large European Liver Transplant Registry analysis, Visseren et al. reported a recurrence rate of 16.7% after a median of five years, accompanied by substantially increased risks of graft loss, mortality, and retransplantation [18]. In contrast, rPSC was less frequent in our cohort, occurring in 8% of patients after RYHJ and in none after D-D reconstruction. This discrepancy should be interpreted with caution, as it is likely attributable to the limited sample size, the single-center design, and the time-dependent nature of rPSC. Given that rPSC frequently develops several years after LT, longer follow-up is required to capture late events and assess whether the lower recurrence rate observed in our cohort is sustained over time. One recipient in the RYHJ group developed cholangiocarcinoma 3.5 years after LT, with metastatic disease at diagnosis. Although this single case cannot support causal inferences, it underscores the broader clinical challenge of timely biliary evaluation in PSC and highlights the practical benefit of easier endoscopic access after D-D reconstruction when postoperative biliary symptoms or imaging abnormalities arise, which may be particularly relevant in PSC recipients at risk of malignant or recurrent biliary disease [9,10].

The central aim of this study was to evaluate biliary complications, as PSC is intrinsically a biliary disease, and postoperative strictures and cholangitis are major contributors to post-transplant morbidity. Consistent with previous studies, the overall incidence of cholangitis and composite biliary complications did not differ significantly between RYHJ and D-D reconstructions, supporting the concept that the reconstruction technique itself is not the primary determinant of biliary morbidity in PSC [15,16,19]. Cholangitis was frequent in both groups, and the time to the first episode of cholangitis did not differ significantly. The observation that cholangitis episodes occurred later in RYHJ recipients should be interpreted cautiously, given the limited sample size and potential differences in diagnostic pathways. While meta-analyses have reported a higher overall risk of ascending cholangitis after RYHJ reconstruction [10], there is currently no robust evidence specifically addressing cholangitis-free survival after LT for PSC, as most studies have focused on composite biliary outcomes or graft survival rather than cholangitis-free survival as a distinct endpoint. Additional studies are needed to determine whether the type of reconstruction influences the timing or recurrence pattern of post-transplant cholangitis in PSC [10,12,13].

In exploratory regression analyses, older recipient age and male donor sex were associated with biliary complications. Recipient age has been repeatedly implicated as a risk factor for biliary and vascular complications after LT, potentially reflecting a more fragile microvasculature, impaired regenerative capacity, and a higher burden of comorbidities [20]. The association between male donor sex and biliary events is less consistent and remains mechanistically uncertain; proposed mechanisms include sex-related differences in bile composition and immune response, although evidence is limited and inconsistent [21,22]. Given the sample size and wide confidence intervals, these associations should be considered hypothesis-generating rather than definitive.

A key strength of this study is the homogeneous PSC cohort managed at a single center with standardized perioperative protocols. This setting enabled a consistent evaluation of outcomes over time during the institutional transition from routine RYHJ to a selective D-D reconstruction strategy. Nevertheless, the present study should be interpreted as confirmatory and implementation-oriented rather than as evidence establishing a new standard of care. Previous systematic reviews and meta-analyses have already demonstrated that D-D reconstruction can be safely performed in appropriately selected PSC recipients [10,19]. The principal contribution of our study is the evaluation of the real-world implementation of a structured, anatomy-driven selection algorithm based on explicit preoperative and intraoperative assessment of recipient bile duct anatomy. By linking the choice of biliary reconstruction to predefined anatomical criteria in routine clinical practice, our findings provide practical evidence on the feasibility and clinical performance of this approach, including its associated spectrum of biliary complications, their management, and long-term outcomes. Overall, our results support the use of D-D anastomosis as the preferred reconstruction technique when recipient anatomy is favorable, while confirming that RYHJ remains an essential alternative for patients with distal bile duct involvement or other anatomical features precluding safe D-D reconstruction. Rather than proposing a new standard of care, these findings reinforce existing evidence and demonstrate that a selective, anatomy-based reconstruction strategy can be successfully implemented in routine clinical practice.

However, several limitations warrant consideration. First, the retrospective design and small sample size reduce statistical power and increase susceptibility to sparse-data effects in multivariate modeling. Second, the anatomy-driven selection strategy (while clinically appropriate) introduces confounding by indication: distal extrahepatic duct involvement differed between groups and may influence both reconstruction choice and biliary outcomes. Third, differences in postoperative diagnostic and therapeutic pathways (particularly endoscopic accessibility) may contribute to differences in detection and management across techniques. These limitations preclude strong causal conclusions regarding the superiority of one reconstruction method over the other.

Most importantly, a critical interpretation of the present data is warranted when considering the presumed practical advantage of D-D reconstruction, namely, preserved endoscopic access. Although the recent ASGE guideline recognizes endoscopic management of biliary complications after both D-D reconstruction and RYHJ, endoscopic access following RYHJ generally requires device-assisted enteroscopy and remains technically more demanding than conventional ERCP after D-D reconstruction [23]. Consequently, preservation of conventional endoscopic access remains a practical advantage of D-D reconstruction. However, this advantage is clinically meaningful only if postoperative biliary complications require endoscopic evaluation or intervention. In our cohort, anastomotic strictures occurred only after D-D reconstruction, whereas no such events were observed after RYHJ. Conversely, bile leakage and surgical revision were more frequent after RYHJ, but these differences did not reach statistical significance. Taken together, these findings do not support a definitive claim of superiority for either reconstruction strategy. Rather, they suggest that the current sample size is too small to draw firm conclusions on clinically relevant trade-offs between accessibility, stricture risk, and leakage-related morbidity. More importantly, the overall burden of biliary complications in both groups remains substantial, underscoring that biliary reconstruction in LT for PSC continues to represent an unresolved clinical problem rather than a settled technical question.

## 5. Conclusions

In this single-center retrospective cohort of PSC recipients undergoing LT, implementation of an anatomy-driven selection strategy for biliary reconstruction was associated with comparable biliary morbidity and similar long-term patient and graft survival after D-D reconstruction and RYHJ. These findings support the feasibility of selective D-D reconstruction when distal bile duct anatomy and duct quality are suitable, while confirming that RYHJ remains essential in patients with unfavorable ductal anatomy. Although the limited sample size precluded definitive statistical significance, the distinct risk–benefit profiles of the two reconstruction techniques are clinically meaningful and reflect routine clinical practice. Given the limited sample size and retrospective design, the results should be regarded as real-world implementation data that are consistent with existing evidence rather than as proof of superiority of either technique.

## Figures and Tables

**Table 1:** Baseline donor and recipient characteristics and intraoperative characteristics stratified by biliary anastomosis technique.

	RYHJ (n = 24)	D-D (n = 22)	*p*-Value
Donor characteristics			
Age [years, mean ± SD]	49.8 ± 14.6	52 ± 18.0	0.653 ^a^
Sex [% male]	50	55	0.777 ^b^
BMI [kg/m^2^, median (Q_0.25_, Q_0.75_)]	26.3 (24.2; 30.7)	25.6 (23.6; 28.8)	0.356 ^c^
ET-DRI [mean ± SD]	1.7 ± 0.4	1.8 ± 0.4	0.561 ^a^
Recipient characteristics			
Age [years, mean ± SD]	43.2 ± 12.0	46.0 ± 12.4	0.540 ^a^
Sex [% male]	88	86	1.000 ^b^
BMI [kg/m^2^, median (Q_0.25_, Q_0.75_)]	24 (22; 26)	24 (21; 25)	0.553 ^c^
MELD score [mean ± SD]	20 ± 12	20 ± 10	0.941 ^a^
Time on waiting list [days, median (Q_0.25_, Q_0.75_)]	474 (203, 744)	245 (98, 1167)	0.655 ^c^
ERCP within 1 year prior to LT [%]	100	86	0.101 ^b^
Placement of biliary stent within 1 year prior to LT [%]	29	18	0.301 ^b^
Age [years, mean ± SD]	43.2 ± 12.0	46.0 ± 12.4	0.540 ^a^
Sex [% male]	88	86	1.000 ^b^
BMI [kg/m^2^, median (Q_0.25_, Q_0.75_)]	24 (22; 26)	24 (21; 25)	0.553 ^c^
MELD score [mean ± SD]	20 ± 12	20 ± 10	0.941 ^a^
Time on waiting list [days, median (Q_0.25_, Q_0.75_)]	474 (203, 744)	245 (98, 1167)	0.655 ^c^
ERCP within 1 year prior to LT [%]	100	86	0.101 ^b^
Comorbid conditions [n, %]			
Autoimmune hepatitis	1 (4)	2 (9)	0.600 ^b^
Incidental Cholangiocarcinoma *	1 (4)	1 (5)	1.000 ^b^
Crohn’s disease	3 (13)	1 (5)	0.609 ^b^
Prior colectomy	2 (8)	2 (9)	1.000 ^b^
Ulcerative Colitis	16 (67)	12 (55)	0.547 ^b^
Intraoperative characteristics			
Cold ischemia time [hours, mean ± SD]	10.7 ± 2.5	10.2 ± 3.6	0.575 ^a^
Warm ischemia time [min, mean ± SD]	40 ± 5	39 ± 12	0.635 ^c^
Duration of Surgery [min, mean ± SD]	486 ± 91	472 ± 120	0.665 ^a^

Data are presented as mean ± standard deviation (SD), median with interquartile range (Q_0.25_, Q_0.75_) or relative frequencies and compared using ^a^ Student’s *t*-test, ^b^ Fisher’s exact test, and ^c^ Mann–Whitney-U-Test. A *p*-value < 0.05 was considered statistically significant. RYHJ, Roux-en-Y hepaticojejunostomy; D-D, duct-to-duct anastomosis; BMI, body mass index; ET-DRI, Eurotransplant donor risk index; MELD, model for end-stage liver disease; ERCP, endoscopic retrograde cholangiopancreatography. * All cholangiocarcinoma were incidental findings in the explanted livers.

**Table 2:** Clinical outcome stratified by biliary anastomosis technique.

	RYHJ (n = 24)	D-D (n = 22)	*p*-Value
Patient survival [%]			0.141 ^d^
30 days	96	95	
90 days	92	95	
1-year	88	95	
3-years	83	91	
5-years	75	91	
Graft survival [%]			0.151 ^d^
30 days	92	91	
90 days	88	91	
1-year	83	86	
3-years	71	82	
5-years	63	77	
ICU Stay [days, median (Q_0.25_, Q_0.75_)]	4 (2, 11)	4 (2, 5)	0.085 ^c^
Hospital Stay [days, median (Q_0.25_, Q_0.75_)]	31 (20, 51)	29 (17, 63)	0.667 ^c^
CCI [mean ± SD]	38 ± 29	43 ± 26	0.545 ^a^
Reoperation [n, %]	9 (38)	6 (27)	0.539 ^b^
Biliary complications [n, %]			
Cholangitis	12 (50)	9 (41)	0.568 ^b^
Anastomotic stenosis	0 (0)	3 (14)	0.101 ^b^
Anastomotic leakage	7 (29)	3 (14)	0.289 ^b^
Anastomotic strictures	0 (0)	3 (14)	0.101 ^b^
Surgical anastomosis revision	7 (29)	3 (14)	0.289 ^b^
rPSC	2 (8)	0 (0)	0.171 ^b^
Non-biliary complications [n, %]			
Hemorrhage	8 (33)	6 (27)	0.484 ^b^
Renal insufficiency	5 (21)	4 (18)	1.000 ^b^
Biopsy-proven rejection	6 (25)	9 (41)	0.348 ^b^
Number of readmissions within 1 year after LT [median (Q_0.25_, Q_0.75_)]	2 (1; 4)	2 (0; 3)	0.463 ^a^
Number of liver-related readmissions within 1 year after LT [median (Q_0.25_, Q_0.75_)]	1 (0; 2)	1 (0; 1)	0.191 ^c^
Re-ERCP within 1 year after LT [n, (%)]	7 (29)	12 (55)	0.134 ^b^
Placement of biliary stent within 1 year after LT [n, (%)]	0 (0)	8 (36)	0.001 ^b^
Re-LT within 1 year after LT [n, (%)]	2 (8)	2 (9)	1.000 ^b^

Data are presented as mean ± standard deviation (SD), median with interquartile range (Q_0.25_, Q_0.75_) or relative frequencies and compared using ^a^ Student’s *t*-test, ^b^ Fisher’s exact test, ^c^ Mann–Whitney-U-Test and ^d^ log-rank Test. A *p*-value < 0.05 was considered statistically significant. RYHJ, Roux-en-Y hepaticojejunostomy; D-D, duct-to-duct anastomosis; ICU, intensive care unit; CCI, Comprehensive Complication Index; rPSC, recurrent primary sclerosing cholangitis; LT, liver transplantation; ERCP, endoscopic retrograde cholangiopancreatography; Re-LT, retransplantation.

**Table 3:** Univariable and multivariable logistic regression analyses of predictors of biliary tract–related complications.

	Univariate OR (95% CI)	*p*-Value	Multivariate OR (95% CI)	*p*-Value
Anastomotic technique (RYHJ vs. D-D)	1.015 (0.318, 3.244)	0.979		
Distal extrahepatic bile duct involvement (yes vs. no)	0.393 (0.109, 1.421)	0.154		
Recipient age (years)	1.052 (0.998, 1.109)	0.060	1.128 (1.031, 1.234)	0.008
Recipient sex (male vs. female)	0.676 (0.111, 4.131)	0.672		
CIT (hours)	0.925 (0.760, 1.126)	0.437		
MELD score	1.045 (0.985, 1.109)	0.144		
ERCP within one year prior to LT (yes vs. no)	0.816 (0.585, 1.138)	0.230		
Placement of biliary stent within one year prior to LT (yes vs. no)	0.540 (0.143, 2.180)	0.386		
Donor age (years)	1.011 (0.974, 1.049)	0.566		
Donor sex (male vs. female)	4.250 (1.234, 14.637)	0.022	11.118 (1.516, 81.538)	0.018
Donor BMI (kg/m^2^)	0.981 (0.876, 1.099)	0.745		
ET-DRI	0.355 (0.072, 1.763)	0.205		

OR, odds ratio; CI, confidence interval; RYHJ, Roux-en-Y hepaticojejunostomy; D-D, duct-to-duct anastomosis; CIT, cold ischemia time; MELD, Model for end-stage liver disease; ERCP, endoscopic retrograde cholangiopancreatography; LT, liver transplantation; BMI, body mass index; ET-DRI, Eurotransplant donor risk index.

## Data Availability

The data presented in this study are available on request from the corresponding author.

## Abbreviations

The following abbreviations are used in this manuscript:

AIH	Autoimmune hepatitis
BDA	Biliodigestive anastomosis
BMI	Body mass index
CCI	Comprehensive Complication Index
CI	Confidence interval
CIT	Cold ischemia time
RYH	Roux-en-Y hepaticojejunostomy
D-D	Duct-to-duct anastomosis
ENIS	Eurotransplant Network Information System
ERCP	Endoscopic retrograde cholangiopancreatography
ET-DRI	Eurotransplant Donor Risk Index
IBD	Inflammatory bowel disease
ICU	Intensive care unit
IQR	Interquartile range
LT	Liver transplantation
MELD	Model for end staged liver disease
MRCP	Magnetic resonance cholangiopancreatography
OR	Odds ratio
PSC	Primary sclerosing cholangitis
rPSC	Recurrent primary sclerosing cholangitis
SD	Standard deviation
WIT	Warm ischemia time
